# High prevalence of fecal carriage of extended spectrum β-lactamase/AmpC-producing *Enterobacteriaceae* in cats and dogs

**DOI:** 10.3389/fmicb.2013.00242

**Published:** 2013-08-16

**Authors:** Joost Hordijk, Anky Schoormans, Mandy Kwakernaak, Birgitta Duim, Els Broens, Cindy Dierikx, Dik Mevius, Jaap A. Wagenaar

**Affiliations:** ^1^Department of Infectious Diseases and Immunology, Faculty of Veterinary Medicine, Utrecht UniversityUtrecht, Netherlands; ^2^Central Veterinary Institute of Wageningen University and Research CenterLelystad, Netherlands

**Keywords:** ESBL, AmpC, companion animals, fecal carriage, *enterobacteriaceae*, cat, dog

## Abstract

Extended-spectrum-β-lactamase (ESBL)/AmpC producing *Enterobacteriaceae* have been reported worldwide amongst isolates obtained from humans, food-producing animals, companion animals, and environmental sources. However, data on prevalence of fecal carriage of ESBL/AmpC producing *Enterobacteriaceae* in healthy companion animals is limited. This pilot study describes the prevalence of ESBL/AmpC encoding genes in healthy cats and dogs, and cats and dogs with diarrhea. Twenty fecal samples of each group were cultured on MacConkey agar supplemented with 1 mg/L cefotaxime and in LB-enrichment broth supplemented with 1 mg/L cefotaxime, which was subsequently inoculated on MacConkey agar supplemented with 1 mg/L cefotaxime. ESBL/AmpC genes were identified using the Check-Points CT103 micro array kit and subsequently by sequencing analysis. Chromosomal *ampC* promoter mutations were detected by PCR and sequencing analysis. From the healthy and diarrheic dogs, respectively 45 and 55% were positive for *Escherichia coli* with reduced susceptibility for cefotaxime. From the healthy and diarrheic cats, the estimated prevalence was respectively 0 and 25%. One diarrheic cat was positive for both reduced susceptible *E.* *coli* and *Proteus mirabilis*. The ESBL/AmpC genes found in this study were mainly *bla*_CTX-M-1_, but also* bla*_CTX-M-14_, *bla*_CTX-M-15_, *bla*_TEM-52-StPaul_, *bla*_SHV-12_, and *bla*_CMY-2_ were detected. This pilot study showed that the prevalence of ESBL/AmpC producing *Enterobacteriaceae* in healthy and diarrheic dogs, and diarrheic cats was relatively high. Furthermore, the genes found were similar to those found in isolates of both human and food-producing animal origin. However, since the size of this study was relatively small, extrapolation of the data to the general population of cats and dogs should be done with great care.

## INTRODUCTION

Extended-spectrum-β-lactamase (ESBL) and plasmid mediated (p)AmpC-producing *Enterobacteriaceae* have been isolated from humans, different animal species, and the environment worldwide. ESBL/AmpC producing *Enterobacteriaceae* in both humans and animals have also been reported increasingly (Coque et al., 2008; Wieler et al., 2011). One of the main concerns is that resistance caused by these enzymes may result in reduced efficacy of antimicrobial therapy or therapy failure. Despite many studies on ESBL/AmpC producing bacteria from different sources, clear data especially on routes of transmission is still lacking. Therefore the epidemiology of ESBL/pAmpC’s is poorly understood. One of the driving forces behind the increased resistance is the use of 3rd and 4th generation cephalosporins in both humans and animals (Dutil et al., 2010; Damborg et al., 2011, 2012). Resistance to these compounds may appear very quickly in case food-producing animals receive antimicrobial treatment resulting in a subsequent increase in resistance in the human population, as was shown in poultry and humans (Dutil et al., 2010). A similar increase was also shown within individual dogs and horses treated with antimicrobials (Damborg et al., 2011, 2012). In companion animals, among others, the 1st generation cephalosporin cephalexin and 3rd generation, long acting cephalosporin cefovecin are commonly used and licensed in Europe. Companion animals have also been suggested as potential reservoir for antimicrobial resistant bacteria (Guardabassi et al., 2004). Several studies have reported the presence of ESBL/pAmpC-producing *Enterobacteriaceae* in clinical samples from companion animals (Schink et al., 2011; Dierikx et al., 2012b; Ewers et al., 2012), however, knowledge about intestinal carriage of ESBL/AmpC’s in healthy companion animals is limited (Costa et al., 2008; Murphy et al., 2009; Gandolfi-Decristophoris et al., 2013). In these studies, all using different isolation methods, the prevalence of *Escherichia coli* with reduced susceptibility to 3rd generation cephalosporins in dogs varied from 0 to 17% and in cats from 0 to 12%. The present study combined the analysis of intestinal carriage of *Enterobacteriaceae* with reduced susceptibility to cefotaxime in both healthy dogs and cats, and dogs and cats with diarrhea.

## MATERIALS AND METHODS

### SAMPLE COLLECTION

Fecal samples were collected from December 2011 to March 2012 from healthy dogs (*n* = 20) and cats (*n* = 20) from different parts of the Netherlands. The samples from the healthy dogs were randomly selected from a longitudinal study on the presence of enteropathogens. Fecal samples from dogs (*n* = 20) and cats (*n* = 20) with diarrhea which were submitted for bacteriological and parasitological examination between December 2011 and February 2012 to the Veterinary Microbiological Diagnostic Center (VMDC) of the Faculty of Veterinary Medicine of Utrecht University were included. A cotton swab was used to inoculate each sample both onto MacConkey agar supplemented with 1 mg/L cefotaxime (MacC+) and in 1 ml LB-medium supplemented with 1 mg/L cefotaxime (LB+) for enrichment. After overnight incubation 10 μl LB+ was plated on MacC+. From samples showing growth on MacC+ without previous selective enrichment, five colonies were selected. From samples showing growth on MacC+ after selective enrichment, one colony was selected.

### SPECIES IDENTIFICATION

Species identification was performed biochemically on all isolates that were selected for further analysis using triple sugar iron (TSI) and ornithine decarboxylase (ODC) and additionally checked for urease production and tryptophan reduction (indole).

### ESBL/AmpC CHARACTERIZATION

To identify ESBL/AmpC producing phenotypes, all selected isolates were screened with a double disk synergy test including cefotaxime (30 μg), cefotaxime with clavulanic acid (30/10 μg), ceftazidime (30 μg), ceftazidime with clavulanic acid (30/10 μg) and cefoxitin (30 μg; Beckton, Dickinson and Company, Breda, Netherlands). Results were interpreted according to CLSI guidelines (CLSI, 2006).

From each animal positive for ESBL/AmpC-producing *Enterobacteriaceae,* one isolate was selected for molecular characterization. If an animal harbored isolates with different phenotypes, based on biochemical analysis and disk diffusion, one isolate of each phenotype was selected. These isolates (*n *= 46) were first screened for the epidemiologically most relevant beta-lactamase genes using the Check-MDR CT-103 tube array according to manufacturers’ protocol (Check-Points, Wageningen, Netherlands). These targets include: *bla*_CTX-M_, *bla*_TEM_, *bla*_SHV_, *bla*_CMY-__ 1/MOX_, *bla*_ACC_, *bla*_DHA_, *bla*_ACT/MIR_, *bla*_CMY-__2/FOX_, *bla*_NDM_, *bla*_KPC_, *bla*_VIM_, *bla*_IMP_, and *bla*_OXA-48_. Subsequently, PCR products of the identified genes were sent for sequence analysis (Macrogen, Amsterdam, Netherlands) using primers previously described (Dierikx et al., 2012b). The online database for ESBL/AmpC genes was used as a reference (; last accessed 10 July 2013) with additional references for *bla*_TEM-52_ variants (GenBank AF126444 and AY883411). Furthermore, sequence analysis of the chromosomal *ampC* promoter region was performed, using primers designed by Caroff et al. (1999). The *ampC* classification was performed as described by Mulvey et al. (2005).

### STATISTICAL ANALYSIS

Confidence intervals for the prevalence estimates were determined by calculating Exact Binomial Confidence Intervals using SAS v9.2 (SAS Institute Inc., NC, USA).

## RESULTS

### SAMPLE COLLECTION AND SPECIES IDENTIFICATION

After direct plating, nine healthy dogs (45%) were positive for cefotaxime reduced susceptible (CTX-RS) *E.* *coli* (**Table 1**). After selective enrichment, no additional dogs were found positive. From the diarrheic dogs, 11 animals were positive for CTX-RS *E.* *coli *(55%), of which eight were positive after direct plating and three more were obtained after selective enrichment. No CTX-RS *Enterobacteriaceae* were found in healthy cats. Four cats with diarrhea (20%) were positive for CTX-RS *E.* *coli* after direct plating and one animal (5%) was positive for both CTX-RS *E. coli* and *Proteus* *mirabilis* (25% in total).

**Table 1:** Prevalence of cats and dogs carrying ESBL/AmpC producing Enterobacteriaceae.

Group	*n* screened	Prevalence % (*n*)	Confidence interval (95 %)
Dogs healthy	20	45.0 (9)	23.1–68.5
Dogs diarrheic	20	55.0 (11)	31.5–76.9
Dogs total	40	50.0 (20)	33.8–66.2
Cats healthy	20	0.0 (0)	0.0–16.8
Cats diarrheic	20	25.0 (5)	8.7–49.1
Cats total	40	12.5 (5)	4.2–26.8

### ESBL/AmpC CHARACTERIZATION

Molecular analysis showed that within each group of animals there was a high variety of ESBL/pAmpC genes present, especially within diarrheic cats (**Table 2**). In this group four out of five animals harbored more than one ESBL/pAmpC gene. The diarrheic cats were positive for *bla*_CTX-M-1_ and _-15_, *bla*_SHV-12_,* bla*_ CMY-2_, and a *bla*_TEM-52-StPaul_ variant. This *bla*_TEM-52__ -StPaul_ variant was identical to the one submitted to GenBank with number AF126444.

**Table 2:** Number of animals carrying ESBL/pAmpC genes in Enterobacteriaceae isolated from healthy cats and dogs, and diarrheic cats and dogs in Netherlands.

	ESBL/AmpC coding gene	Dog		Cat	
		Healthy	Diarrheic	Healthy	Diarrheic
No. of animals carrying	*bla*_CTX-M-1_	4	2	–	–
	*bla*_CTX-M-15_	–	–	–	1
	*bla*_CTX-M-14_	–	1	–	–
	*bla*_CTX-M-1_ +* bla*_CTX-M-14_	–	1	–	–
	*bla*_CTX-M-1_ +* bla*_TEM-52-StPaul_	–	–	–	1
	*bla*_CTX-M-1_ + *bla*_CMY-2_	–	–	–	1
	*bla*_CTX-M-1_ +* bla*_CMY-2_ +*ampC* type-3	–	–	–	1^f^
	*bla*_CTX-M-1_ +* bla*_CMY-2_ + * bla*_TEM-35_	–	1^a^	–	–
	*bla*_CTX-M-1_ +* bla*_CMY-2_ + unknown^b^	1	–	–	–
	*bla*_CTX-M-1_ +* ampC *type-3	–	1	–	–
	*bla*_SHV-12_ + *bla*_CMY-2_	–	–	–	1
	*bla*_CMY-2_	2	2	–	–
	*ampC* type-3^c^	1	1	–	–
	*ampC* type-new^d^	0	1	–	–
	Unknown^b^	1	1	–	–
	>1 R gene^e^	1	3	0	4
	*E. coli*	9	11	0	4
	*E. coli *+* P. mirabilis*	0	0	0	1^f^

a This animal carried one *E. coli* harboring both a *bla*_TEM-35_ gene and a *bla*_CMY-2_ gene. The *bla*_CTX-M-1_ gene and another *bla*_CMY-2_ gene were found in separate *E. coli*.

b Isolates negative on Check-MDR CT-103 array, and no *ampC* type-3.

c Classification as previously described (Mulvey etal., 2005).

d New *ampC* variant compared to previously described variants (Mulvey etal., 2005).

e Number of animals with more than one type of ESBL/pAmpC gene.

f This animal carried a *P. mirabilis* harboring a *bla*_CMY-2_ gene. All other genes reported in this table were found in *E. coli*.

Diarrheic dogs were positive for *bla*_CTX-M-1_, _ -__ 1__ 4_, *bla*_TEM-35_, and *bla*_CMY-2_ genes, of which three animals harbored more than one type of ESBL/AmpC (**Table 2**). The strain containing *bla*_TEM-35_, which is an inhibitor resistant TEM (Zhou et al., 1994), also harbored *bla*_CMY-2_. An AmpC phenotype was shown for this strain by a double disk synergy test.

Healthy dogs were positive for *bla*_CTX-M-1_ and *bla*_CMY-2_ genes (**Table 2**). One healthy dog harbored three ESBL/pAmpC determinants, respectively *bla*_CTX-M-1_, *bla*_CMY-2_, and an isolate with reduced susceptibility to cefotaxime in which no resistance gene was found. Further molecular typing was performed by sequence analysis of the chromosomal *ampC* promoter region.

Healthy dogs, diarrheic dogs, and diarrheic cats all harbored the *ampC*-type-3 variant (Mulvey et al., 2005). Chromosomal *ampC*-types 4, -5, -7, -11, -12, and -18 were also found, however, based on disk diffusion results, these variants showed ESBL as well as AmpC phenotypes, depending on the other ESBL/pAmpC gene present in the same isolate. Therefore the contribution of these chromosomal mutations to resistance could not be confirmed. One diarrheic dog harbored an *ampC* type-wt and one new *ampC* variant. The new *ampC* variant shows close resemblance to *ampC* type-2 (Mulvey et al., 2005), with a substitution of cytosine to guanine at position +20. Disk diffusion results showed this variant had an AmpC phenotype. No other ESBL/AmpC genes were found in this isolate. Furthermore, two healthy dogs were negative in the array and harbored either a wild-type *ampC* or *ampC* type-11 gene. Results from the disk diffusion assay were inconclusive. Because reduced cefotaxime susceptibility caused by the isolate only harboring an *ampC *wild-type or type-11 could not be confirmed phenotypically, these three isolates from the healthy and diarrheic dog were designated “unknown” (**Table 2**).

## DISCUSSION

In the animals included in this study there is a high level of intestinal carriage of ESBL/AmpC-producing *Enterobacteriaceae* in healthy dogs (45%), and both diarrheic dogs (55%) and cats (25%). In all animal groups in most fecal samples large numbers of reduced susceptible *E. coli* or *P. mirabilis* were found after direct plating on selective MacConkey agar plates. Only few fecal samples were found positive additionally after inoculation using the more sensitive selective enrichment and subsequent pure culturing on selective MacConkey agar. Even though the proportion of ESBL/AmpC producing *E. coli*/*P. mirabilis* as part of the total population of *E. coli*/*P. mirabilis* present in the fecal sample was not determined, this suggests that the bacterial cell count of ESBL/AmpC producing *E. coli*/*P. mirabilis* in these fecal samples is relatively high.

Due to the small size of the study, the confidence intervals for the estimated prevalence were relatively large (**Table 1**). Despite this fact, the prevalence in the different animal groups, especially for both healthy and diarrheic dogs, observed in the present study was relatively high compared to other studies. Costa et al. (2008) showed in a study on healthy cats (*n *= 36) and dogs (*n *= 39) that two *E. coli* isolates, both from the same dog, were reduced susceptible to cefotaxime, indicating a prevalence of 2.6%. The fact that no selective culturing media were used in that study may contribute to this large difference. In healthy cats they did not find isolates with reduced susceptibility to cefotaxime. Murphy et al. (2009) reported that in two regions insouthern Ontario 0% of both cats (*n *= 39) and dogs (*n *= 188) were positive for *E. coli* with reduced susceptibility to cefotaxime. In that study, fecal samples were also screened using selective media. Gandolfi-Decristophoris et al. (2013) showed that in Switzerland from thefecal swabs that were taken from healthy cats (*n *= 202) and dogs (*n *= 174) at nursing homes and at veterinary clinics, 17% of the examined dogs and 12% of the cats were positive for *Enterobacteriaceae* with reduced susceptibility for the 3rd generation cephalosporin cefpodoxime. In contrast 2.9% of the dogs and 2% of the cats examined were positive for ESBL genes. This difference in prevalence between cefpodoxime resistance and presence of ESBL genes may be caused by the fact that the presence of AmpC type genes was not determined. As we have shown, *bla*_CMY-2_-like genes were present in more than 10% of all examined isolates. Additionally, we have also shown that promoter mutations in the chromosomal *ampC* gene were present.

Gandolfi-Decristophoris et al. (2013) also indicated that antimicrobial treatment (not further specified) that was administered in the three months prior to the investigation was identified as a risk factor for the carriage of ESBL producing *Enterobacteriaceae*. Similar results were shown by Damborg et al. (2011) in a study in which cefalexin was orally administered to dogs. This treatment resulted in selection for the presence of *bla*_CMY-2_ producing *E. coli* in the feces. The treatment history of the animals included in our study is not known. Therefore, any possible contribution of previous antibiotic usage as described above could not be established.

In China, a more or less similar prevalence was observed compared to our study (Sun et al., 2010). In their data set 93% of the samples were fecal samples. From the healthy animals included in their study, 24.5% (26 out of 109) were positive for ESBL producing bacteria. From the sick animals 54.5% (73 out of 134) were positive for ESBL producers. This prevalence was based on a double disk synergy test on isolates obtained from various animal clinics.

The sample size in the present study (20 animals per category) was relatively small. Therefore extrapolation of the results toward the general population of cats and dogs in the Netherlands should be done with great care. The comparisons made to other studies are regarded appropriate since these studies included sample sets of more or less comparable size (Murphy et al., 2009; Damborg et al., 2011; Gandolfi-Decristophoris et al., 2013).

The resistance genes reported in this study are similar to those found in both humans and food-producing animals (EFSA, 2011; Ewers et al., 2012). The predominant resistance gene reported in the present study is *bla*_CTX-M-1_. The high abundance of this gene is consistent with studies on isolates from humans and farm animals in the Netherlands, in which *bla*_CTX-M-1_ is either the most or second most predominant gene (Dierikx et al., 2012a; Voets et al., 2012; Hordijk et al., 2013; Platteel et al., 2013). However, more data on plasmids and host bacteria are required to draw any conclusions on genetic relatedness and possible transmission to or from either humans or food-producing animals. Furthermore, little is known about persistence of ESBL/AmpC carriage and about the significance of shedding of ESBL/AmpC producing bacteria by companion animals into their environment. This requires additional research.

We conclude that despite the estimated prevalence of ESBL/AmpC producing *Enterobacteriaceae* especially in both healthy and diarrheic dogs, but also diarrheic cats is relatively high. They are certainly a reservoir for the resistance genes. Whether pets are a source for humans cannot be determined by this observational study, but this reservoir should certainly be included in attribution studies for human infections. Furthermore, using selective media to isolate ESBL/AmpC producing bacteria may reduce the chance to underestimate the prevalence of these bacteria.

## Conflict of Interest Statement

The authors declare that the research was conducted in the absence of any commercial or financial relationships that could be construed as a potential conflict of interest.
